# Death of Count Taaffe

**Published:** 1896-03

**Authors:** 


Death of Count Taaffe. On November 29, 1895, Count Taaffe died after long suffering, and in him a man has departed whose great knowledge and remarkable executive ability places him in the group of Austria's first statesmen. His personal amiability and kindness have placed a monument in the hearts of everyone who ever came in contact with him. The veterinary surgeons of Austria have Count Taaffe to thank as the reorganizer of the whole Austrian sanitary and veterinary system, so that the long epoch in which he was active as the head of the

Upper Sanitary Department in Austria forms a turning point in the history of Austrian veterinary science. May, therefore, all honor be given to his memory.—*Thierärzt Centralblatt*, December 15, 1895.

